# Ongoing repair of migration-coupled DNA damage allows planarian adult stem cells to reach wound sites

**DOI:** 10.7554/eLife.63779

**Published:** 2021-04-23

**Authors:** Sounak Sahu, Divya Sridhar, Prasad Abnave, Noboyoshi Kosaka, Anish Dattani, James M Thompson, Mark A Hill, Aziz Aboobaker

**Affiliations:** 1 Department of Zoology, University of OxfordOxfordUnited Kingdom; 2 CRUK/MRC Oxford Institute for Radiation Oncology, ORCRB Roosevelt Drive, University of OxfordOxfordUnited Kingdom; Max Planck Institute for Biology of AgeingGermany; California Institute of TechnologyUnited States

**Keywords:** regeneration, stem cells, cell migration, Planarian

## Abstract

Mechanical stress during cell migration may be a previously unappreciated source of genome instability, but the extent to which this happens in any animal in vivo remains unknown. We consider an in vivo system where the adult stem cells of planarian flatworms are required to migrate to a distal wound site. We observe a relationship between adult stem cell migration and ongoing DNA damage and repair during tissue regeneration. Migrating planarian stem cells undergo changes in nuclear shape and exhibit increased levels of DNA damage. Increased DNA damage levels reduce once stem cells reach the wound site. Stem cells in which DNA damage is induced prior to wounding take longer to initiate migration and migrating stem cell populations are more sensitive to further DNA damage than stationary stem cells. RNAi-mediated knockdown of DNA repair pathway components blocks normal stem cell migration, confirming that active DNA repair pathways are required to allow successful migration to a distal wound site. Together these findings provide evidence that levels of migration-coupled-DNA-damage are significant in adult stem cells and that ongoing migration requires DNA repair mechanisms. Our findings reveal that migration of normal stem cells in vivo represents an unappreciated source of damage, which could be a significant source of mutations in animals during development or during long-term tissue homeostasis.

## Introduction

Constant threats to genome integrity by both exogenous and endogenous agents has led to the evolution of cellular mechanisms to counteract various forms of DNA damage (Hoeijmakers, 2001; Tubbs and Nussenzweig, 2017). Accumulated genotoxic damage, particularly in stem cells, is thought to underpin both ageing and the development of cancer (Vitale et al., 2017; Mandal et al., 2011; Goodell and Rando, 2015; Jackson and Bartek, 2009). Therefore, maintaining the integrity of the genome is particularly important for longer-lived animals with adult stem cells. We know that efficient DNA repair mechanisms to counteract the effects of replicative stress and other sources of damage are central to avoiding both premature ageing and cancer (Rossi et al., 2008; Jeggo et al., 2016; Sperka et al., 2012; Adams et al., 2015). Recent in vitro studies using cancer cell lines, dendritic cells, as well as primary stem cells have shown that migration through micro capillaries or extreme constrictions imparts mechanical stress on nuclei, and this can be a source of DNA damage and genome instability (Denais et al., 2016; Raab et al., 2016; Irianto et al., 2017a; Smith et al., 2019; Nader, 2020; Shah et al., 2017; Shah, 2020; Kirby and Lammerding, 2018). These studies reveal an assortment of mechanisms by which mechanical stress results in DNA damage. This damage can be either repaired in a regulated manner or cells may undergo differentiation after being reimplanted into animals after in vitro manipulation (Smith et al., 2019). However, evidence of damage caused by purely in vivo adult stem cell migration and whether it is a significant load to DNA repair mechanisms is currently conspicuously absent. If migrating cells, in particular stem cells, experience DNA damage in vivo, this has a number of broad implications for metazoan development and homeostasis. For example, migrating stem cells may experience aberrant effects on stem cell differentiation, become transformed to cause malignancies, or become senescent and contribute to ageing all as a result of DNA damage (Smith et al., 2019). As a corollary of this, we would expect that active DNA repair mechanisms might be required to allow continued cell migration in vivo.

However, it is not known whether migration and damage are associated in vivo in normal adult stem cells, as to this point cells have been manipulated to experience migration through artificial constrictions in vitro. To address this, we have improved on existing methods to study DNA damage and repair (DDR) processes in the highly regenerative animal *Schmidtea mediterranea*, focusing on the observation of migrating adult stem cells (Abnave et al., 2017; Guedelhoefer and Sánchez Alvarado, 2012). We used an established assay in which migrating stem cells can be observed and with which we previously established that stem cells home precisely to wounds, form cell extensions when migrating, and require the transcription factors snail and zeb1, which regulate epithelial to mesenchymal transition, for migration. (Abnave et al., 2017; Guedelhoefer and Sánchez Alvarado, 2012). Investigating the DNA damage response in this context, we found that the adult stem cell population displays increased levels of DNA damage as they migrate towards a distal wound site and repair this damage when they reach the wound site. We demonstrate that both the repair of DNA damage and active DNA repair mechanisms are required to allow directed stem cell migration to a wound site. Overall, our data find in vivo evidence for the link between cell migration and DNA damage and suggest that a reconsideration of the significance of migratory events across development and adult homeostasis in the context of potential DNA damage is required.

## Results and discussion

### A robust DNA damage response allows stem cells to resist doses up to 15 Gy of ionising radiation

**Figure 1.**
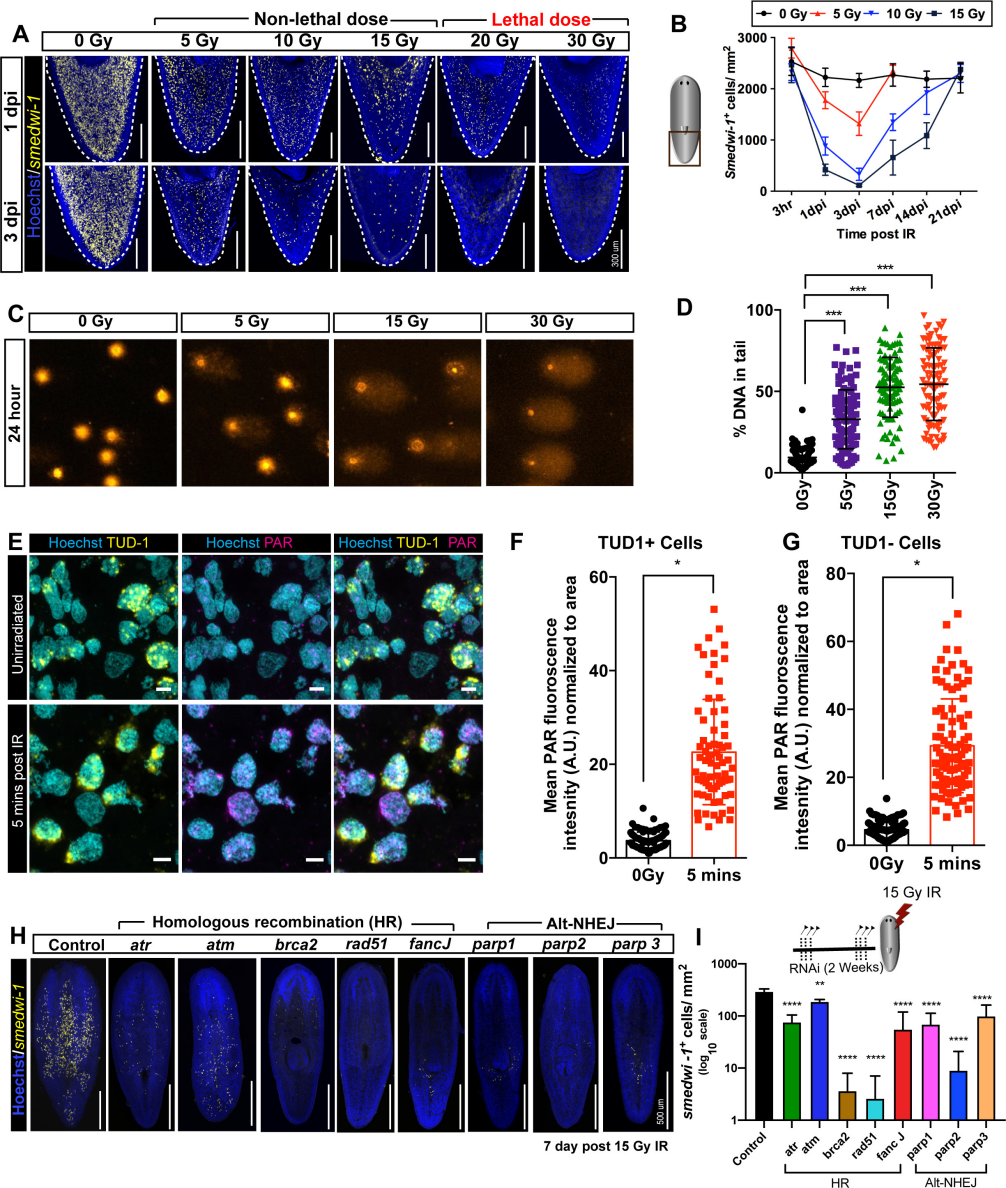
Planarian stem cell resistance to doses up to 15 Gy of gamma IR requires conserved DDR pathways. (**A**) smedwi-1 FISH of planarians exposed to different doses of gamma IR (5, 10, 15, 20, and 30 Gy) after 1 and 3 days post-IR (dpi) showing a dose-dependent decrease in stem cell number. Scale bar: 300 μm. (**B**) Quantification of smedwi-1^+^ cells/mm^2^ (yellow) showing the repopulation kinetics of surviving stem cells after different doses of IR post-IR (n = 5 per dose, per time point). Results are expressed as mean ± SD. (**C**) COMET assay showing the extent of DNA breaks (comet shape) in isolated planarian cells at 24 hr after exposure to 5, 15, and 30 Gy of IR. (**D**) Quantification of the percentage of tail DNA in COMET assay post-IR at 24 hr. Results are expressed as mean ± SD. Each dot represents the tail DNA in individual planarian cells. (***p<0.0001, one-way ANOVA using Tukey’s multiple comparison test). (**E**) Double immunostaining with Anti-TUD-1 (Yellow) and Anti-PAR (Magenta) showing DNA damage in stem cells (Tud-1^+^) and post-mitotic differentiated cells (Tud-1^−^) at 5 min post 5 Gy IR. Nucleus is stained with Hoechst (blue). (**F–G**) Quantification of PAR fluorescence in Tud1^+^ and Tud1^−^ cells normalised to the nuclear area in irradiated and unirradiated cells (0 Gy) (*p<0.0001. Student’s t-test). (**H**) Representative FISH showing stem cell repopulation in Control (gfp) RNAi and after knockdown of different DNA repair genes (involved in homologous recombination [atr, atm, brca2, fancJ, rad51] and Alt-NHEJ [parp1, parp2, parp3]) after 7 days post 15 Gy IR. Gene name represents the RNAi condition. (**I**) Repopulation of smedwi-1^+^ cells/mm^2^ in DDR RNAi worms after 7 days post 15 Gy IR (n = 5 per condition). Results are expressed as mean ± SD in log_10_ scale (***p<0.0001, **p<0.001, one-way ANOVA using Tukey’s multiple comparison test). Figure 1—source data 1.Numerical data used to make Graphs B, D, F, G, and I.

Planarian flatworms have a population of pluripotent adult stem cells, and potentially avoid both ageing and cancer (Sahu et al., 2017; Rink, 2013; Wagner et al., 2011). When exposed to 20 and 30 Gray (Gy) lethal doses of ionising radiation (IR), some *smedwi1*^+^ stem cells remain after 24 hr but are not competent to proliferate and rescue the animal. On the other hand, all animals survived exposure to a 15 Gy dose, as amongst the surviving *smedwi1*^*+*^ stem cells some are competent to proliferate and differentiate normally (Figure 1A,B, Figure 1—figure supplement 1A–D). Stem cell loss after sub-lethal irradiation is dose dependent and continues until 3 days post-irradiation (pi) (Figure 1B).

**Figure 1—figure supplement 1.**
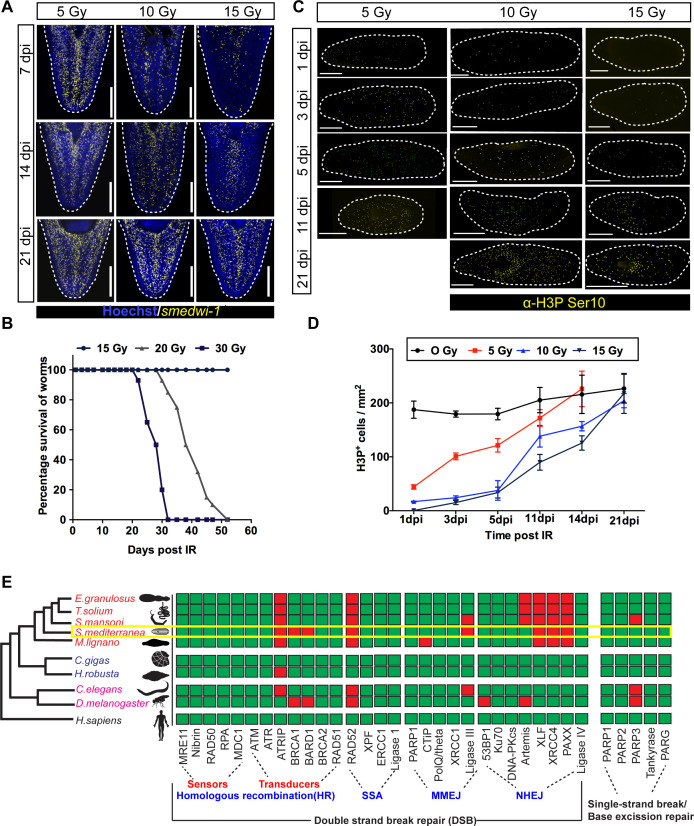
Dynamics of stem cell proliferation and repair kinetics of DNA damage in planarian stem cells. (**A**) FISH showing repopulation of smedwi-1^+^ stem cells after different doses of IR at indicated days post-IR (dpi). Scale bar: 300 μm. (**B**) Survival curve indicating the percentage of animals alive after exposure to different doses of ionising radiation, n = 15 per dose. Unirradiated and worms exposed up to 15 Gy IR showed 100% survival. (**C**) Immunostaining with mitotic marker Anti-H3 phosphorylated-ser10 (H3pSer10) showing the repopulation of mitotic cells (yellow) after exposure to different doses of gamma IR (5, 10, 15 Gy) at indicated days post-IR (dpi). Scale: 500 μm. (**D**) Quantification shows the repopulation kinetics of mitotic cells at different doses of irradiation (n = 5 per condition). Results are expressed as mean ± SD. (**E**) The presence (green) or absence (red) of conserved DNA repair genes involved during double-stranded break repair (DSB) as sensors and transducers in homologous recombination, single-stranded annealing (SSA), microhomology-mediated end-joining (MMEJ), and non-homologous end-joining (NHEJ), single-stranded break repair in metazoans. The yellow box highlights the DNA repair proteins in *S. mediterranea.* The phylogenetic tree is based on Grohme et al., 2018. Figure 1—figure supplement 1—source data 1.Numerical data used to make Graphs B and D.

**Figure 1—figure supplement 2.**
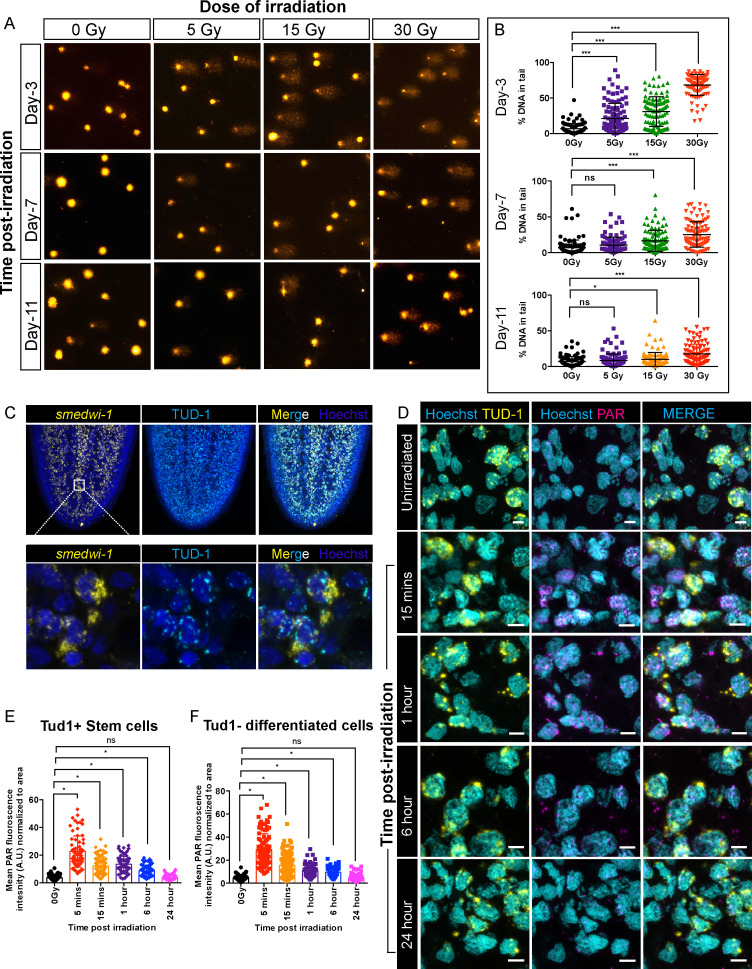
Detection of DNA breaks and DNA damage response in planarian cells after irradiation. (**A**) COMET assay showing the extent of DNA breaks (Comet-shaped tails) on dissociated planarian cells at 3, 7, and 11 days post-exposure to 5, 15, and 30 Gy of gamma IR. (**B**) Quantification of the percentage of tail DNA in individual comet at indicated time points and dose of IR. (n = 100 comets per condition/time point) (*p<0.0001, Tukey’s multiple comparison test). (**C**) Smedwi-1 FISH and Tudor-1 (TUD1) immunostaining showing the presence of perinuclear chromatoid bodies (TUD1^+^, Cyan) in smedwi-1^+^ stem cells (Yellow). Nucleus is stained with Hoechst (Blue). (**D**) Double immunostaining with Anti-TUD1 (yellow) and Anti-Poly ADP Ribose (PAR, magenta) antibodies showing increase in nuclear PAR formation in irradiated planarian cells compared to unirradiated controls; scale bar: 10 µm. (**E, F**) Quantification of PAR fluorescence normalised to the area of nucleus from individual TUD1^+^ stem cells (**E**) and TUD1^−^ differentiated cells (**F**) at 5 min, 15 min, 1 hr, 6 hr, and 24 hr post-exposure to 5 Gy IR. The nucleus is stained with Hoechst and pseudo-coloured to cyan. The perinuclear TUD1 staining and Hoechst stained nucleus is used to measure the area of individual nuclei. (*p<0.0001, ANOVA using Tukey’s multiple comparison test). Figure 1—figure supplement 2—source data 1.Numerical data used to make Graphs B, E, and F.

**Figure 1—figure supplement 3.**
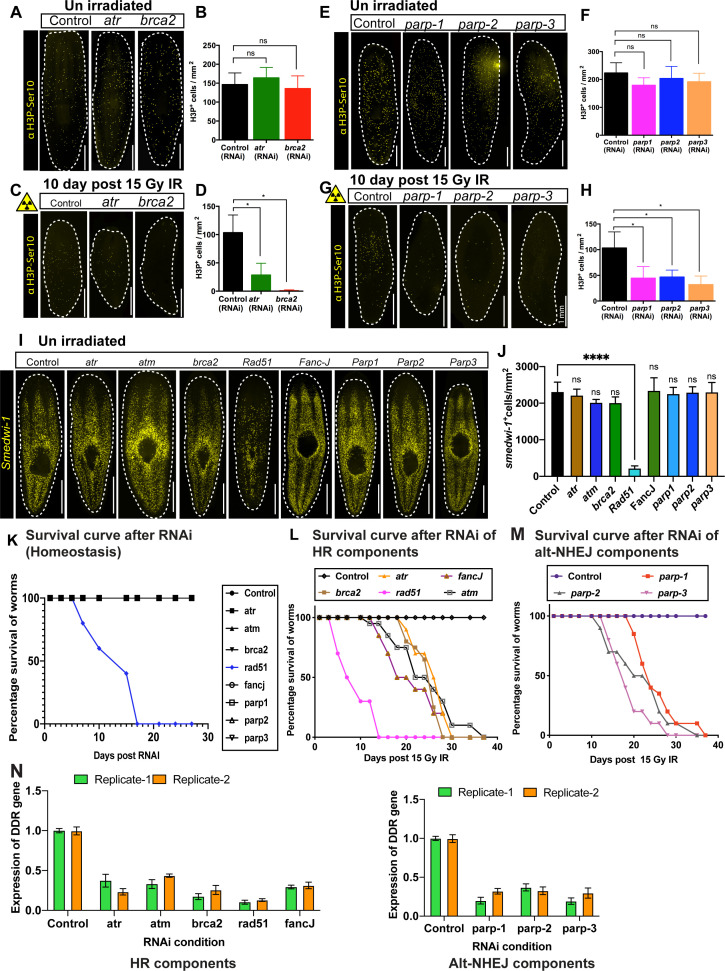
Role of DNA repair gene in planarian stem cell maintenance. (**A**) Immunostaining with H3pSer10 shows the presence of mitotic cells in control worms and after atr and brca2 RNAi. (**B**) Quantification of H3P^+^ cells show no significant difference in cell division after atr and brca2 RNAi. (n = 5 per RNAi). Results are expressed as mean ± SD and (p=0.6442 [*atr*], p=0.8506 [*brca2*], ns = not significant, Tukey’s multiple comparison test). (**C**) Immunostaining with H3pSer10 showing the repopulation of mitotic cells in control worms and after atr and brca2 RNAi worms, 10 days post 15 Gy IR. (**D**) Quantification of H3p^+^ cells shows a significant difference in the repopulation of mitotic cells after atr and brca2 RNAi after irradiation, 10 days post 15 Gy IR. (n = 5 per RNAi). Results are expressed as mean ± SD (*p<0.0001, Tukey’s multiple comparison test). (**E**) Immunostaining with H3pSer10 shows the presence of mitotic cells in control worms and after parp1, parp2, and parp3 RNAi. (**F**) Quantification of H3p^+^ cells shows no significant difference in cell division after parp1, parp2 and parp3 RNAi. (n = 5 per RNAi) Results are expressed as mean ± SD and Student’s t-test used for analysis (p=0.2279 [*parp1*], p=0.8074 [*parp2*], p=0.5001 [*parp3*], ns = not significant, Tukey’s multiple comparison test). (**G**) Immunostaining with H3pSer10 showing the repopulation of mitotic cells in control worms and after parp1, parp2, and parp3 RNAi worms, 10 days post 15 Gy IR. (**H**) Quantification of H3p^+^ cells show a significant difference in the repopulation of mitotic cells after *parp1, parp2*, and *parp3* RNAi after irradiation. (n = 5 per RNAi) (*p<0.0001, Tukey’s multiple comparison test). (**I**) Representative FISH showing stem cell density in Control (gfp) RNAi and after knockdown of different DNA repair genes [involved in homologous recombination (atr, atm, brca2, fancJ, rad51) and Alt-NHEJ (parp1, parp2, parp3)] at unirradiated/homeostatic condition. (**J**) Quantification of smedwi-1^+^ cells/mm^2^ in DDR RNAi worms (n = 3–5 per condition). Results are expressed as mean ± SD (***p<0.0001, ns = not significant, one-way ANOVA using Tukey’s multiple comparison test). (**K**) Survival curve of DDR RNAi worms showing lethality of Rad51 RNAi worms in homeostatic condition (n = 10 worms per condition). (**L, M**) Survival curve of DDR RNAi worms after exposed to 15 Gy IR (n = 15 worms per condition). (**N**) qPCR results showing the efficiency of knockdown of DNA repair genes. Figure 1—figure supplement 3—source data 1.Numerical data used to make Graphs B, D, F, H, J, K, L, M, and N.

We optimised the use of the COMET assay and staining with antibodies to poly-ADP ribose (PAR), the currently available methods for measuring DNA damage/repair in planarians (Shibata et al., 2016; Wouters et al., 2020; Peiris et al., 2016), to quantify DNA damage in planarian cells. We observe that damage assayed by single-cell gel electrophoresis of whole planarian stem cell populations is IR dose dependent (Figure 1C,D), with repair and reduction in COMET signal taking place over the subsequent 11 days (Figure 1—figure supplement 2A,B). By combining PAR staining with an antibody to planarian Tudor-1 that marks the perinuclear RNP granules (chromatoid bodies) (Solana et al., 2009) in smedwi1^+^ stem cells (Figure 1—figure supplement 2C), we measured damage in stem cells and post-mitotic differentiated cells simultaneously using total nuclear PAR. Levels of PAR staining in the nucleus increased just 5 min after exposure to 5 Gy ionising radiation (IR) (Figure 1E–G) and returned to the baseline within 24 hr after exposure (Figure 1—figure supplement 2D–F), indicating that this approach accurately measures acute DNA damage events.

We identified conserved DDR genes from the *S. mediterranea* genome and transcriptome (Grohme et al., 2018; Brandl et al., 2016; Robb et al., 2008; Figure 1—figure supplement 1E) that are known to be essential elsewhere to repair assorted DNA breaks. The transcripts of most DNA repair genes (*atr*, *atm*, *brca2*, *parp1*, and *parp2*) are enriched in stem cells based on the expression pattern data from sorted stem cells and stem cell progeny and from single-cell expression data (Dattani et al., 2018; Wurtzel et al., 2015). We found that RNAi of conserved DDR genes individually (*atr*, *atm*, *brca2*, *fancJ*, *parp1*, *parp2*, *parp3*) during normal regeneration and homeostasis did not lead to any phenotypic defects in regenerating animals and did not affect stem cell number or proliferation over a time course of several weeks (Figure 1—figure supplement 3I–J). Only RNAi of rad51 led to animal death as previously reported (Shibata et al., 2016; Figure 1—figure supplement 3I–J). These data suggest that RNAi of these individual genes does not disrupt normal stem cell function during homeostasis. This may reflect the incomplete effects of RNAi, compensation between repair pathways (Figure 1—figure supplement 3N), or both.

After sub-lethal IR exposure, surviving stem cells clonally expand to restore homeostatic and regenerative capacity in a dose-dependent manner (Wagner et al., 2011; Wagner et al., 2012; Lei et al., 2016; Figure 1A,B, Figure 1—figure supplement 1A–D). In this scenario, RNAi of the individual conserved components of homologous recombination (HR) (*atr*,* atm*, *brca2*, *rad51*, and *fancJ*) and alt-non-homologous end-joining pathways (NHEJ) (*parp1*, *parp2*, and *parp3*) after 15 Gy IR led to a failure in stem cell repopulation (Figure 1H,I, Figure 1—figure supplement 3A–H). As well as establishing robust methods for measuring DNA damage our data provide proof of principle that well-known DDR genes have an ongoing role in stem cell survival, and in DNA repair after IR exposure. This establishes a basis for using *S. mediterranea* as an experimentally tractable in vivo model for studying DDR in adult stem cells. It remains unclear if the lack of phenotypic effect after RNAi of individual DDR genes during normal homeostasis and in the absence of extensive external genotoxic stress is due to incomplete knockdown (Figure 1—figure supplement 3N) or compensation between repair mechanisms, or if longer term RNAi experiments over months/years may eventually result in defects with repair machinery has reduced efficiency, but we did not investigate this possibility.

### Migrating stem cells undergo migration-coupled DNA damage that resolves at the wound site

Having established robust DNA damage assays in planarians, we next wished to understand if we could observe whether stem cell migration leads to DNA damage in vivo, as has been observed for mammalian cells constricted in vitro. Planarian stem cells and their progeny must migrate to the site of a wound or during reproductive (asexual) fission to form a regenerative blastema (Reddien and Sánchez Alvarado, 2004; Wenemoser and Reddien, 2010). Recent work using cells in culture has shown that mechanical stress on the cell nucleus, through a variety of proposed mechanisms, can lead to DNA damage and genome instability during cell migration (Denais et al., 2016; Raab et al., 2016; Shah, 2020; Lomakin et al., 2020; Irianto et al., 2017b; Bennett et al., 2017). However, how important this is generally in vivo in animals is unknown. To study this phenomenon in planarians, despite the lack of live-cell imaging approaches, we established a robust assay for stem cell migration (Abnave et al., 2017). This uses a lead shield to perform ‘shielded irradiation’ and obtain a stripe of stem cells whose subsequent migration can be followed (Figure 2—figure supplement 1A–E). Head amputation triggers anterior migration from the shielded strip of stem cells towards the wound (Figure 2—figure supplement 1C) and a lack of posterior cell migration over the experimental time course allows us to clearly define the posterior and therefore the pre-migration anterior boundary of the shield. This system has already allowed the detailed study of planarian stem cell migration in vivo (Abnave et al., 2017), for example demonstrating that migrating stem cells develop cytoplasmic projections, precisely home to small ‘poke’ wound sites and require the activity of transcription factors with conserved roles in epithelial to mesenchymal transition.

**Figure 2.**
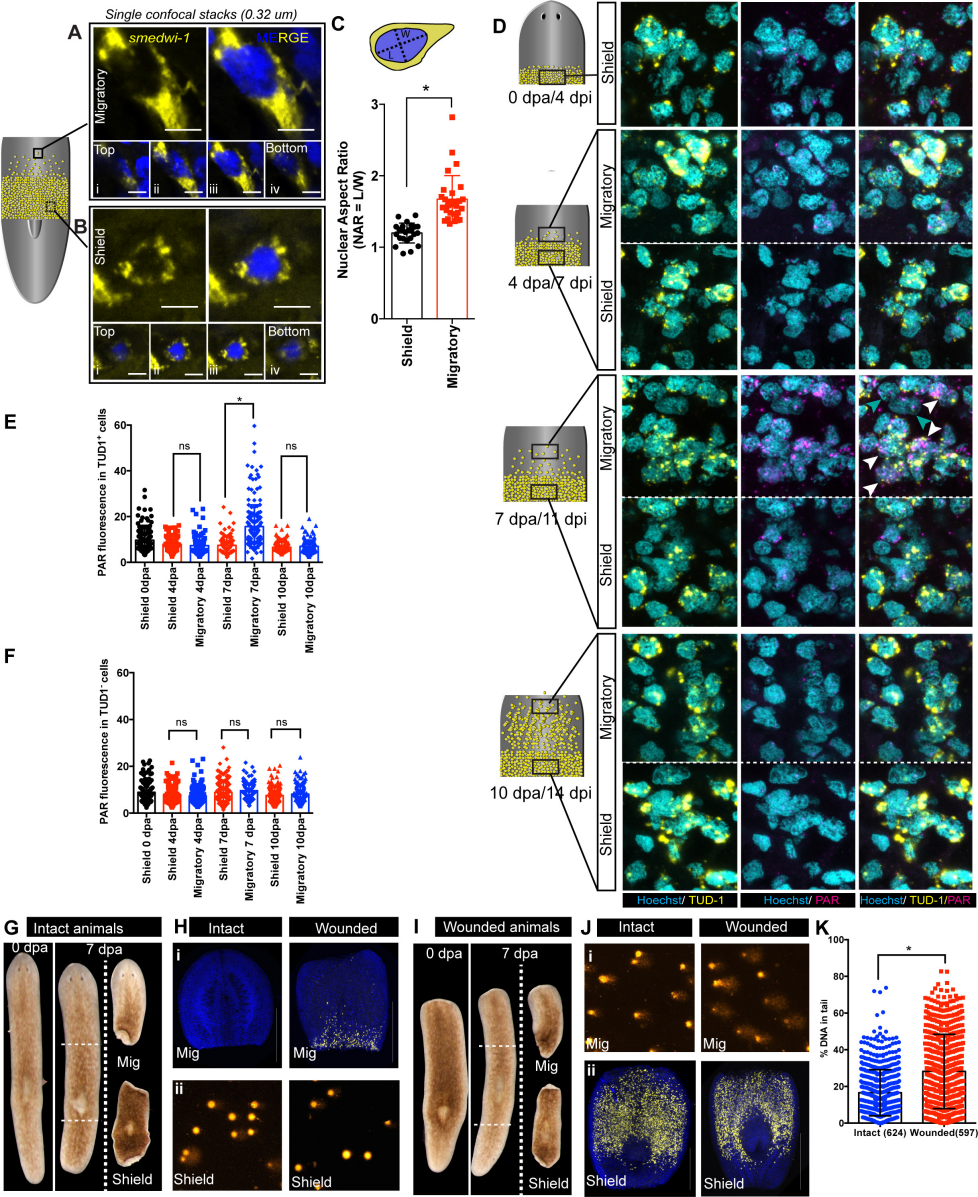
Migration-coupled DNA damage (MCDD) in stem cells. Representative FISH showing stem cells (*smedwi-1^+^*) with extended protrusions in migratory cells (**A**) and stationary cells from the shielded region (**B**). Nuclei stained with Hoechst (blue). Images are shown as single confocal Z-stack (0.32 μm). (i–iv) is the single Z-stack from top to bottom. Scale bar: 5 μm. The cartoon explains the setup of shielded irradiation assay where a lead shield is placed in the middle and a lethal dose of 30 Gy is given to these worms. Cells under the shield (Yellow) are protected from IR and starts to migrate after amputation. (**C**) Quantification of nuclear aspect ratio (NAR) in the migratory cells compared to stationary cells (n = 28 cells in migratory region and n = 24 cells from the shield at 7 dpa/11 dpi [shielded irradiation assay]) (*p<0.0001. Student’s t-test). (**D**) Immunostaining with anti-PAR (magenta) in migrating stem cells (anti-TUD-1, yellow) after 0, 4, 7, and 10 days post-amputation showing MCDD in TUD-1^+^ migrating stem cells. Box denotes the field of cells imaged for analysis. Nuclei stained with Hoechst (cyan). Scale bar: 5 μm. White arrows denote increased nuclear PAR staining in Tud1^+^ stem cells at 7 dpa, and gray arrow denotes lack of PAR fluorescence in Tud1^−^ cells. Quantification showing the PAR fluorescence normalised to the nuclear area from Tud1^+^ stem cells (**E**) and post-mitotic differentiated Tud1^−^ cells (**F**) in the migrating region compared to stem cells in the shield. The measurement of PAR fluorescence is strictly nuclear and results are expressed as mean ± SD. (*p<0.0001; one-way ANOVA using Tukey’s multiple comparison test). Brightfield images of intact (**G**) and wounded (**I**) animals at 0 dpa and 7 dpa showing the amputated migratory region and shielded region. Dotted lines denote the position of the shield. The migratory region was used for *smedwi-1* FISH and the corresponding shielded region was used for COMET assay and vice versa depending on the context (refer to Figure 2—figure supplement 3). (**H**) *smedwi-1* FISH of the migratory tissues at 7 dpa showing the presence of migrating stem cells in wounded animals compared to no migration in intact animals. Cells corresponding to the shielded region were used for COMET assay to check for the extent of DNA damage. (**J**) *smedwi-1* FISH of the shielded tissue at 7 dpa showing the presence of stem cells under the shield in intact and wounded animals. Cells corresponding to the migratory region from the animals were used for COMET assay to check for the extent of DNA damage in migrating stem cells in wounded animals. (**K**) Quantification of COMET assay showing the extent of DNA breaks in migrating cells in wounded animals compared to intact animals (absence of migrating stem cells). Each dot represents the percentage of tail DNA from single cells after COMET assay (n = 624 cells from intact animals, and 597 cells in wounded animals). Results are expressed as mean ± SD (student’s t-test; *p<0.0001). Figure 2—source data 1.Numerical data used to make Graphs C, E, F, and K.

Using this assay, we asked if normal stem cell migration in vivo leads to increased DNA damage. Planarian stem cells are characterised by large nuclear-to-cytoplasmic ratios like other animals stem cells (Aboobaker, 2011; Gehrke and Srivastava, 2016), suggesting that the nucleus in migrating stem cells could encounter physical stress through deformation of normal nuclear shape. In order to check nuclear shape plasticity in migrating cells compare to stationary cells, we measured the nuclear aspect ratio (NAR) (Chen et al., 2015) of the cells in the migratory region compared to stationary cells at 7 day post-amputation and observed significant changes in NAR (Figure 2A–C, Figure 2—figure supplement 1F–G). Planarian stem cells in the migratory region showed increased NAR (ranging from 1.7 to 2 or higher) (Figure 2—figure supplement 1F–G). Consistent with our findings, a recent study using mutant skeletal stem cells also reported that a distribution of NAR ranging from 1.7 to 1.9 induces DNA damage (Earle et al., 2020). The magnitude of the change in NAR in migrating planarian stem cells suggested that, by analogy with mammalian stem cells in culture (Earle et al., 2020), there was potential for mechanical forces on the nucleus that could cause DNA damage.

**Figure 2—figure supplement 1.**
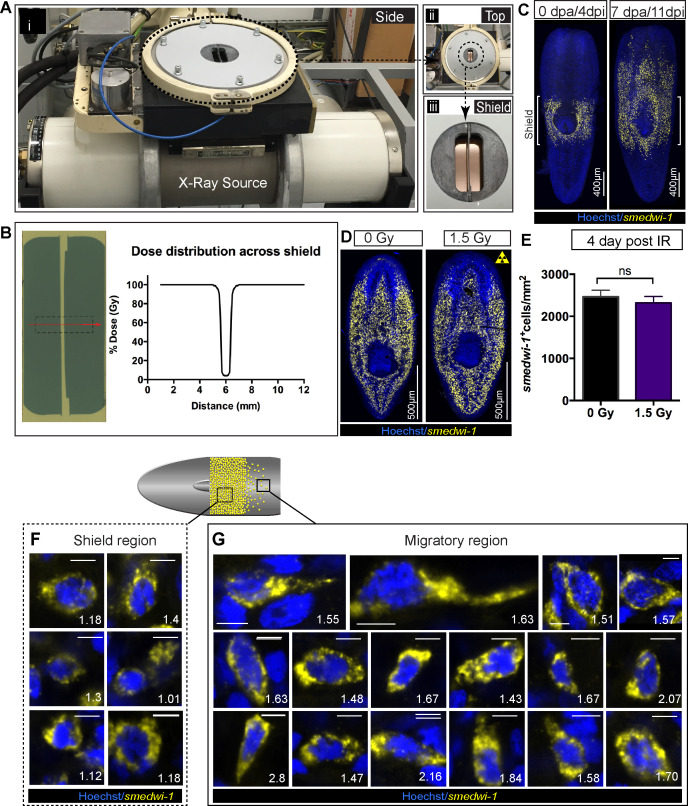
Shielded irradiation assay to study stem cell migration and change in nuclear aspect ratio in migrating cells. (**A**) Experimental set up showing the shielded irradiation assay (i), with a top view showing the position of the shield (ii) and focusing on the shield (iii). (**B**) Dose distribution across the lead strip showing greater than 95% attenuation of X-ray (Abnave et al., 2017). An exposure of 30 Gy corresponds to a dose to the cells directly above the shield-protected region of less than 1.5 Gy. (**C**) Representative FISH of smedwi-1 showing the distribution of stem cells (yellow) after shielded irradiation assay and the migration of stem cells from the shield after 7 day post-head amputation. Brackets ‘[]’ represents the position of the shield. Scale bar = 400 μm. (**D**) Representative smedwi-1 FISH showing survival of stem cell after 4 day post 1.5 Gy of IR. (**E**) Graph represents smedwi-1^+^ cells/mm^2^ showed no significant difference in stem cell maintenance after 1.5 Gy IR. (n = 3, p=0.3148, Student’s t-test). (**F–G**) Representative FISH showing stem cells (smedwi-1^+^) with extended protrusions in migratory cells (**F**) and stationary cells from the shielded region (**E**). Nuclei stained with Hoechst (blue). Images are shown as single confocal Z-stack (0.32 μm). Number represents the NAR value of individual cells, plotted in Figure 2C. Scale bar = 5 μm. Figure 2—figure supplement 1—source data 1.Numerical data used to make Graph E.

**Figure 2—figure supplement 2.**
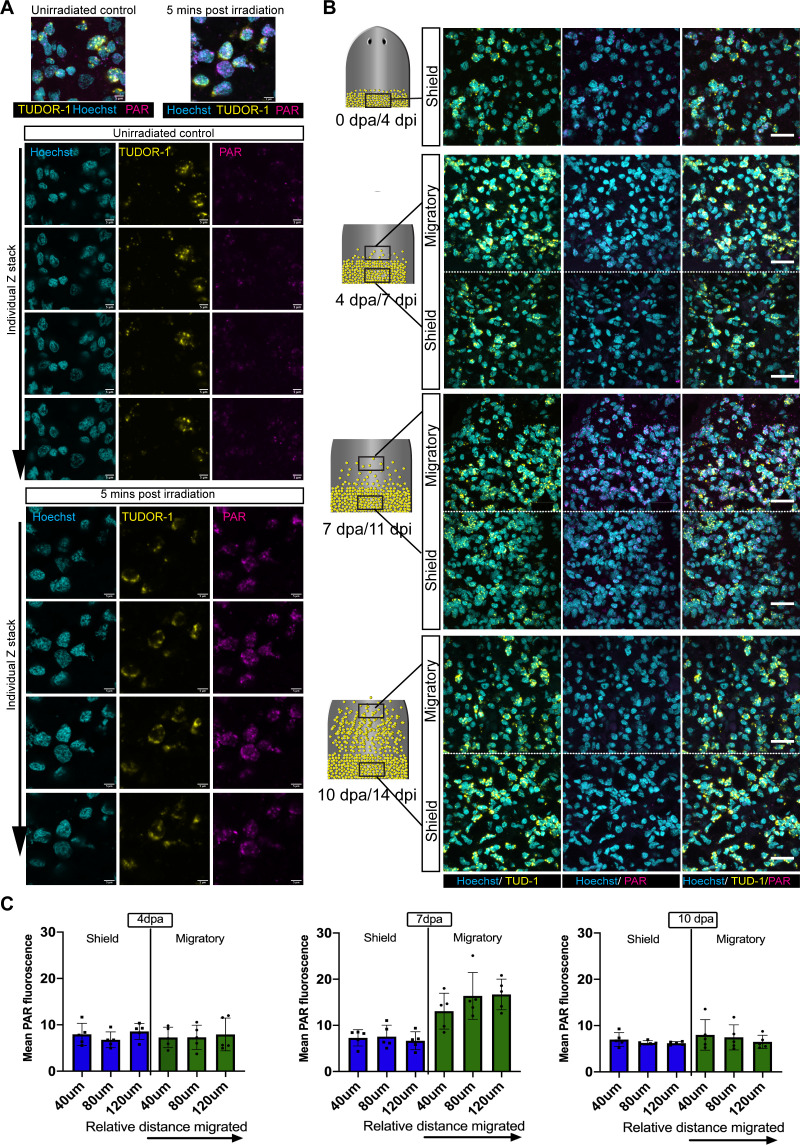
Migration coupled DNA damage in stem cells. (**A**) Maximum projection of individual nucleus showing the localisation of nuclear PAR (magenta) surrounded by perinuclear Tudor one staining (yellow). The nucleus is stained by Hoechst (cyan). Single confocal z stacks of the representative image clearly show the Tud-1 staining to be perinuclear and evidence of strong nuclear PAR expression in the nucleus (after IR exposure) in all the stacks. The nuclear outline is measured based on the Hoechst channel, and total PAR fluorescence was then measured and normalised to the area. Nuclei is stained with Hoechst (cyan), scale bar: 5 µm. (**B**) Zoomed out images of Tud-1^+^ stem cells showing expression of PAR in the migratory region as compared to shielded region (related to Figure 2D). (**C**) Measurement of DNA damage response in Tud-1^+^ stem cells with distance travelled during migration. PAR fluorescence in stem cells at a 4 dpa, 7 dpa, and 10 dpa in the shielded irradiation assay within 40 µm slices along the anterior posterior axis starting at the most anterior cell and going back towards the shield. We observed an increase in PAR fluorescence at 7 dpa as cells migrate further away from the shield. This increasing trend of damage along the gradient is not evident in the shielded region, or at 10 days when cells have reached the wound site and stoppe migrating. Each dot represents the average of the total number of cells present in each 40 µm section within each worm (n = 5). Figure 2—figure supplement 2—source data 1.Numerical data used to make Graph E.

**Figure 2—figure supplement 3.**
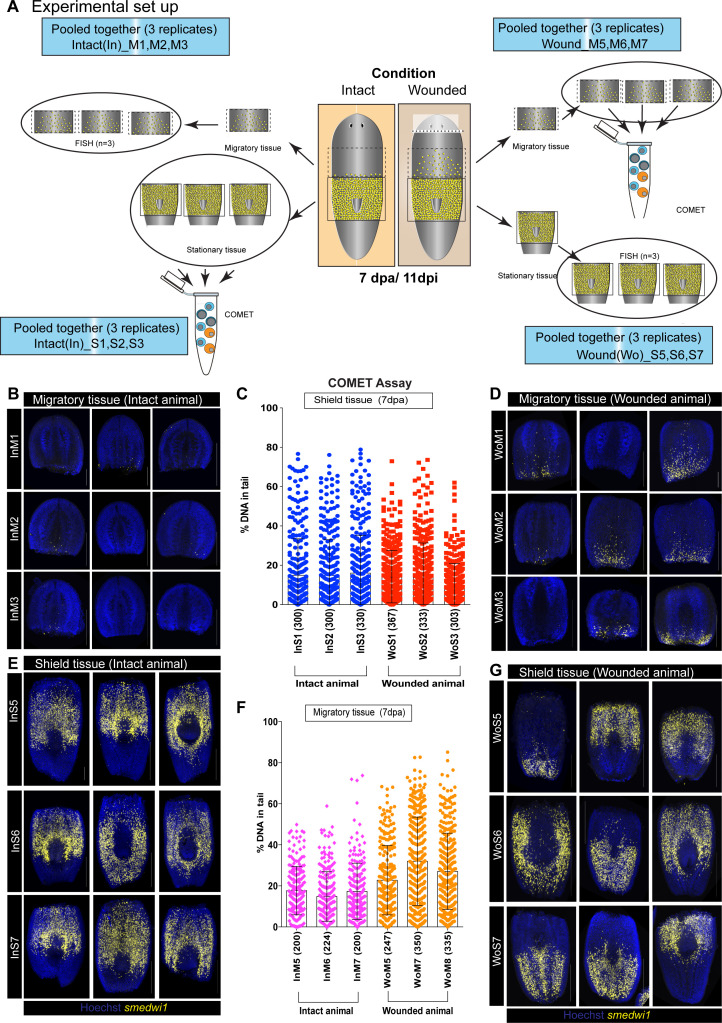
COMET assay to detect DNA breaks during stem cell migration. (**A**) A schematic showing the experimental strategy to perform COMET assay coupled with smedwi-1 FISH to accurately measure DNA breaks in shielded tissue and migratory tissue. Planarian worms were amputated (Wounded) 4 days after the shielded irradiation assay and at 7 day post-amputation (7 dpa/11 dpi) the shielded tissue and the migratory tissue were amputated. Individual migratory tissue fragments from intact and wounded animals were used for COMET assay and the corresponding shielded tissue were used for smedwi-1 FISH and vice verse. Each FISH is performed in three batches (InM1, InM2, InM3) with three worms per batch. The panels are named as ‘In’ or ‘Wo’ corresponding to Intact or Wounded. ‘M’ and ‘S’ correspond to Migratory tissue or Shielded tissue. The numbers denote different batch of experiment. The corresponding shielded tissue from these worms were pooled for COMET assay. (**B, D**) smedwi-1 FISH from migratory tissue of intact animals showing no migration compared to migrating smedwi-1^+^ stem cells in wounded animals (**D**). (**C**) Graph represents the percentage of DNA in tail from individual comets from cells in the shielded tissue from intact and wounded animals (300 comets were analysed per condition, n = 900 comets from intact animals and n = 900 from wounded animals). (**E, G**) smedwi-1 FISH from shielded tissue of intact (**E**) and wounded animals (**G**) showing the accuracy of cutting the migratory region. The tip of the tail was also amputated before the COMET assay/FISH to reduce the number of dead cells from the irradiated tissue. (**F**) Graph represents the percentage of DNA in the tail of individual comets from cells in the migratory tissue from intact and wounded animals showing an increase in DNA breaks in cells from the migratory region compared to the shielded region. Numbers in parenthesis depicts the total number of comets analysed. The data from (**F**) is used in Figure 2L. Figure 2—figure supplement 3—source data 1.Numerical data used to make Graph E.

We then performed anti-PAR/TUD-1 double immunostaining (Shibata et al., 2016), to test whether the changes in nuclear shape in migrating stem cells lead to increased levels of DNA damage. We found that migrating TUD-1^+^ cells accumulate increased levels of acute DNA damage with increased migratory distance (Figure 2D,E, Figure 2—figure supplement 2B,C) that eventually return to baseline levels when stem cells reach the wound site, are distributed through the tissue and cease migrating (Figure 2E). We note that the migratory regions and wound site have been subjected to the same conditions in the context of shielded irradiation, so damage is not due to the previous exposure of these regions to IR as acute DNA damage, measured by PAR staining, is not observed when migration stops at the wound site.

Stationary stem cells remaining in the shield do not have increased levels of PAR staining, implicating cell migration as the cause of increased DNA damage (Figure 2D,E). Similarly, post-mitotic TUD1^−^ cells (i.e. not stem cells) that are also present in the migratory region and wounded region environment have lower levels of detectable DNA damage than migrating stem cells (Figure 2F). This suggests that migrating adult stem cells, which have increased NARs capable of inducing DNA damage, experience ongoing DNA damage events while they migrate and until they reach the wound site and stop migrating.

While PAR staining measures an acute regulatory response to repair new damage, a COMET assay measures global levels of DNA breaks. To further validate that the increased DNA damage observed in stem cells in the migratory region, we performed COMET assays on cells in the shielded and migratory regions of the migration assay (Figure 2—figure supplement 3A). We used both intact animals (no migration of stem cells) and animals at 7 days post-amputation (wounded, where stem cell migration is induced). This allowed direct comparison of cell populations from the migratory regions with and without migrating stem cells (Figure 2G–K, Figure 2—figure supplement 3A). We used in situ hybridisation to *smedwi-1* in animal fragments not used for COMET to confirm the accuracy of separating the shielded and migratory regions (Figure 2H,J, Figure 2—figure supplement 3B–G). These experiments revealed that the levels of COMET were increased in migratory regions containing stem cells compared to migratory regions from intact animals that are devoid of stem cells (Figure 2K). We conclude that migrating stem cells have increased levels of DNA strand breaks while they migrate. These experiments provide an independent measure of DNA damage and also show that migrating stem cells have increased levels of DNA damage.

Overall, these experiments to measure NAR, levels of nuclear PAR, and levels of COMET signal demonstrate planarian stem cells undergo migration-coupled-DNA-damage (MCDD) in vivo. Multiple factors have been shown to lead to increased DNA damage, for example nuclear deformation including changes in the localised concentration of DNA repair factors (Bennett et al., 2017) or due to increased replication fork stalling without any disruption of the nuclear envelope (Shah, 2020). A reasonable assumption based on our data and in vitro studies suggests that the change in nuclear shape during migration is connected to increased DNA damage in planarian stem cells. While further work requiring the ability to manipulate planarian cells in vitro and perform live imaging will be needed to demonstrate this link more directly, we nonetheless found that stem cell migration in vivo leads to DNA damage in these cells.

### Stem cells pre-loaded with DNA damage incur a delay in migration

**Figure 3.**
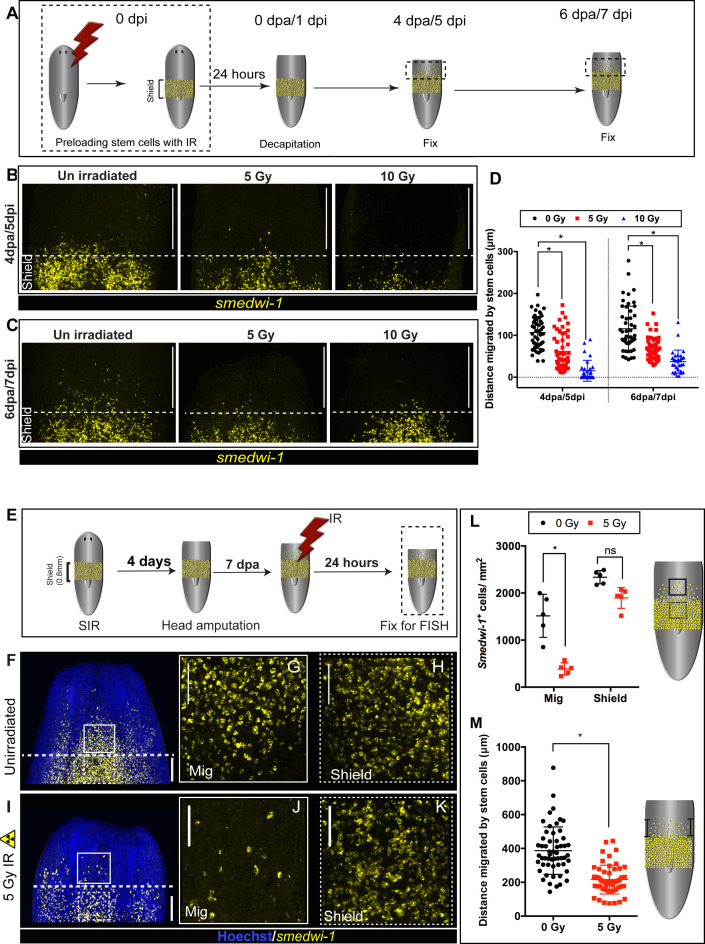
DNA damage delays migration and migrating stem cells with MCDD are more sensitive to ionising radiation. (**A**) Experimental scheme showing worms pre-exposed to irradiation (5 and 10 Gy) followed by a shielded irradiation and amputation after 24 hr. Worms are fixed at 4 dpa and 6 dpa (dpa = days post-amputation). Box represents the migratory region, represented in the figure below. (**B, C**) FISH showing worms pre-exposed to IR (5 and 10 Gy) show delayed stem cell migration after 4 and 6 dpa. Dotted line represents anterior boundary of the shield. Scale bar: 350 μm. (**D**) Distance migrated by 10 most distant cells are counted from individual worms (n = 5 per condition). Results are expressed as mean ± SD. Statistical significance determined by multiple t-test using the Holm–Sidak method, *p<0.05. (**E**) Schematic of experimental set up to study sensitivity of migrating cells to IR. In addition to the initial shielded irradiation, the worms were irradiated with a low dose of IR (5 Gy, whole body) when MCDD is high (7 dpa) and are fixed after 24 hr to check the survival of the migratory stem cells to IR. (**F–K**) Representative smedwi-1 FISH showing migrating cells are more sensitive to IR than the cells in the shielded region. The region counted for analysis is marked with a box (bold: migratory region, dotted: shielded region). (n = 5 per condition, scale bar: 200 μm **F, I;** ;100 μm **G**, **H**, **J**, **K**). (**L**) Quantification of smedwi-1^+^ cells/mm^2^ cells in the shielded region and in the migratory region. The decrease in cells/ mm^2^ in the migratory field is significant compared to the decrease in the shielded region indicating that MCDD sensitises cells to IR. Cartoon showing the region counted for analysis. Each dot represents number of surviving cells from individual worms, n = 5. Statistical significance determined by two-way ANOVA using Tukey’s multiple comparison test (*p<0.05). (**M**) Distance migrated by stem cells showing that cells are more sensitive to low-dose IR the further they have migrated. Each dot represents the distance migrated by individual cells. Distance migrated by 11 most distant cells are counted from individual worms (n = 5 per condition). Results are expressed as mean ± SD (student’s t-test; *p<0.0001, ns = not significant). Figure 3—source data 1.Numerical data used to make Graphs D, L, and M.

We wished to understand whether stem cells in planarians continue to migrate irrespective of DNA damage or whether they repair damage during this process. We hypothesised that stem cells with accumulated DNA damage might pause to remove the source of damage while repairing damage, and then migrate once again. In order to understand, whether the presence of DNA damage acts to inhibit active stem cell migration in vivo, we pre-irradiated whole worms with 5 and 10 Gy IR before following stem cell migratory behaviour in the shielded irradiation assay (Figure 3A, Figure 3—figure supplement 1A). In these experiments, cells in the shielded region have incurred IR-induced DNA damage. We amputated animals to induce migration within 24 hr to trigger stem cell migration while we knew that DNA damage is still present in these cells (Figure 1—figure supplement 2B). We observed that pre-irradiated stem cells undergo a significant delay in migration (Figure 3B–D). Although there is a significant delay in migration, stem cells eventually reach the wound site, maintain normal stem cell numbers, and fuel normal regeneration (Figure 3—figure supplement 1B). These data suggest a relationship between active migration and levels of DNA damage, with increased levels of damage inhibiting active migration until sufficiently repaired. However, irradiation in this experiment also leads to reduction in cell density and cell proliferation in the shield post-IR exposure, and we cannot exclude that the observed delay in migration may be impart due to these factors (Pfeifer et al., 2018).

**Figure 3—figure supplement 1.**
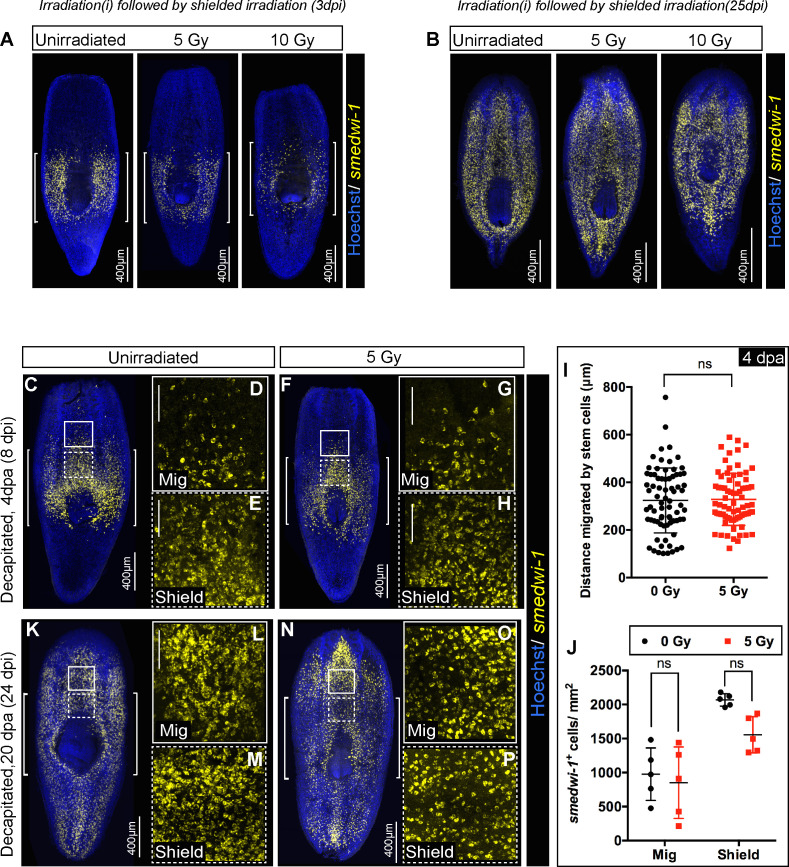
Stem cells pre-loaded with damage before wounding show delays in migration. (**A**) Representative FISH showing the distribution of stem cells in the shield after 5 and 10 Gy of IR followed by the shielded irradiation assay (3 day post-IR). [] brackets indicate the shielded area. Scale bar: 400 μm. (**B**) The stem cells can eventually migrate and rescue the whole animal. Representative FISH showing the stem cells migrate and reach the wound and rescue the worm (25 day post-IR). Scale bar: 400 μm. (**C–P**) Worms are irradiated with a low dose of IR (5 Gy) when cells start to migrate (4 dpa, **C–H**) and cells reach the wound (20 dpa, **K–P**). Worms are fixed after 24 hr to check the survival of the migratory stem cells. Representative smedwi-1 FISH showing the sensitivity of migrating cells and cells in the shielded region. The region counted for analysis is marked with a box (bold: migratory region, dotted: shielded region). (n = 5 per condition). (**I**) Distance migrated by stem cells suggests no significant sensitivity of stem cells to IR (at least 11 distant cells are counted from individual worms and plotted in the graph. Results are expressed as mean ± SD (p=0.48, ns = not significant, student’s t-test) n = 5 worms/condition. (**J**) Quantification of smedwi-1^+^ cells/mm^2^ cells (yellow) in the shielded region and in the migratory region. Rate of decrease in the migratory field and in the shielded region is plotted in the graph. The region counted for analysis is marked with a box (bold: migratory region **G**, **J**, **M**, **P**; dotted: shielded region **H**, **K**, **N**, **Q**). Cells are counted by making a 300 × 300 box anterior to the shield (counted as migratory field) and 300 × 300 box posterior to the shield (counted as shielded region). Results are expressed as mean ± SD and Tukey’s multiple comparison test is used to check for significance. Each dot corresponds to smedwi-1^+ ^cells/mm^2^ in individual worm, n = 5, *p<0.05, ns = not significant). Figure 3—figure supplement 1—source data 1.Numerical data used to make Graphs I and J.

### Stem cells with MCDD show increased sensitivity to IR

Next, we examined whether stem cells with MCDD are more sensitive to IR. If this were the case, it would demonstrate that the increased damage observed during migration is a significant load on the repair machinery. In addition to performing shielded irradiation, the worms were given an additional dose of 5 Gy to the whole animal at 7 days post-amputation (dpa) (Figure 3E), a time point when the highest number stem cells are actively migrating (Abnave et al., 2017) and when the cells have highest levels of MCDD as measured by levels of nuclear PAR (Figure 2E). We observed a significant decrease in stem cell survival in the migratory region after 5 Gy IR compared to stationary stem cells in the shield (Figure 3F–M), demonstrating that migrating stem cells with MCDD are more sensitive to irradiation than stationary cells (Figure 3H). We also found that those cells that had migrated furthest, but had not yet reached the wound site, were the most sensitive to IR (Figure 3M), suggesting that the accumulation of MCDD may, on average, increase with migratory distance from the wound. We did not observe this striking difference earlier in the migratory process (Figure 3—figure supplement 1C–J) when cells have just started migrating in response to wounding, or when cells had already reached the wound site, and migration was complete and MCDD has been repaired (Figure 3—figure supplement 1K–P). This demonstrates that increased radiation sensitivity correlates with increased acute levels of DNA damage, dependent on the extent of active stem cell migration (Figure 2D-E).

### Wound-induced stem cell migration requires active DNA repair mechanisms to resolve ongoing MCDD

**Figure 4.**
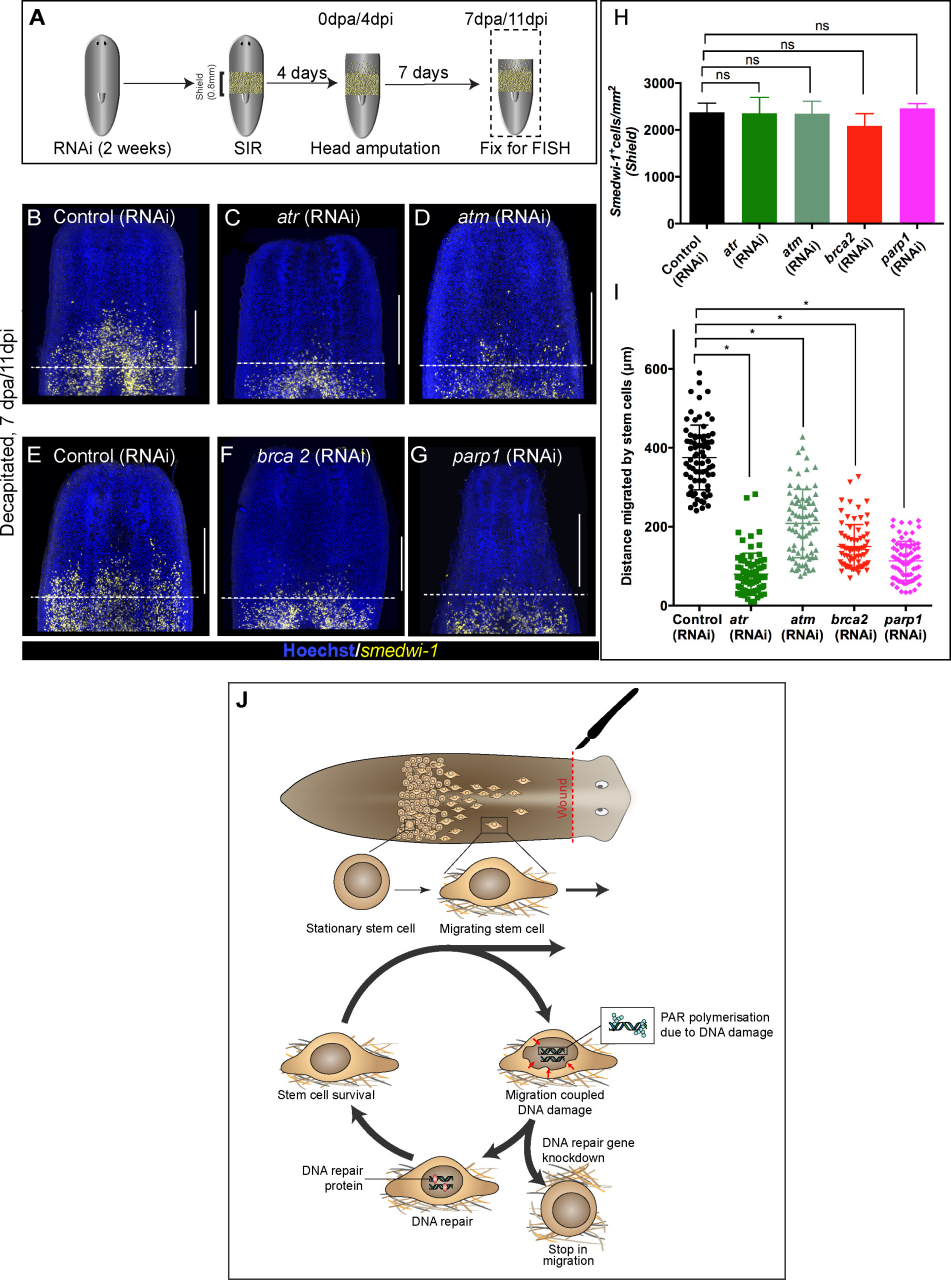
Wound-induced stem cell migration requires active DNA repair mechanisms to resolve ongoing MCDD. (**A**) Experimental scheme to study the role of DDR genes in stem cell migration. Worms are injected for 2 weeks (RNAi) followed by the shielded irradiation assay and fixed for FISH 7 days post-head amputation. (**B–G**) Representative smedwi-1 FISH shows migration of stem cells (yellow) at 7 dpa in control (RNAi) (**B and E**) worms, but migration is inhibited in atr (**C**), atm (**D**) brca2 (**F**), and parp1 (**G**) RNAi worms. (n = 5 per RNAi condition). Scale bar: 400 μm, dotted line represents the anterior boundary of the shielded region. (**H**) Stem cells in the shielded region show no significant changes in the stem cell turnover. (*p<0.05, ns = not significant, p>0.9999 [*atr*], 0.9818 [*atm*], 0.99997 [*brca2*], 0.3722 [*parp1*], respectively), (n = 5 per RNAi condition, Tukey’s multiple comparison test). (**I**) Quantification showing the distance travelled by stem cells after knockdown of DNA repair genes compared to the control RNAi. Each dot represents the distance migrated by individual cells. Distance migrated by 15 most distant cells are counted from individual worms. Results are expressed as mean ± SD n = 75 cells; n = 5 worms/RNAi condition (Tukey’s multiple comparison test; *p<0.0001, ns = not significant). (**J**) Stem cells undergo changes in nuclear shape during migration compared to stationary cells in the shield. This model proposes that stem cells undergo migration, followed by MCDD and DNA repair. In the absence of functional DNA repair machinery stem cells fail to migrate. Figure 4—source data 1.Numerical data used to make Graphs H and I.

We next asked whether active DNA repair pathways are a functional requirement for continued stem cell migration as they are for recovery from non-lethal doses of IR exposure. We therefore performed RNAi of specific DDR genes in the context of the stem cell migration assay (Figure 4A). We observed significantly less migration in *atr*, *atm*, *brca2*, and *parp1* RNAi worms compared to control RNAi worms (Figure 4B–I). We did not observe any significant difference in stem cell density in the shielded region (Figure 4H), suggesting that knockdown of these genes does not affect normal homeostatic stem cell turnover, as we previously observed (Figure 1—figure supplement 3I). The significant reduction in the distance migrated by stem cells (Figure 4I) supports a role for active ongoing DNA repair in maintaining genomic integrity during migration and allowing migration to proceed in the face of ongoing damage to the genome. Migrating stem cells incur DNA damage and then either die or differentiate in the context of DDR component knockdowns, similar to the effects of IR exposure. This is supported by the finding that a dose of 5 Gy, which usually removes 40–50% of stationary stem cells after 24 hr (Figures 1A,B and 3E–M), is sufficient to remove over 80% of migrating stem cells.

In future, the development of live-cell imaging and antibodies detecting upstream DNA damage response factors rather than downstream markers of damage foci dependent on repair pathway activity (like PAR staining) will allow us to track cells and observe if stem cells after DDR component RNAi accumulate DNA damage and eventually die due to failure to repair DNA breaks.

### Conclusions

We have demonstrated that a previously under-appreciated role of DDR is to combat MCDD during stem cell migration in vivo. In the absence of fully functioning DDR machinery, planarian adult stem cells fail to migrate to the wound site in the shielded irradiation assay. Our findings confirm that migration leads to DNA damage in vivo in normal stem cells and, in the light of earlier in vitro findings in cell lines (Denais et al., 2016; Raab et al., 2016; Irianto et al., 2017a; Nader, 2020) and mesenchymal stem cells (Smith et al., 2019), may represent an evolutionarily conserved cost of this process. An ever growing number of studies using cells in culture or cells constricted in vitro and reimplanted into animals tissues suggest that cell migration mechanically impacts cell nuclei, with effects from complete nuclear rupture at one extreme to moderate nuclear deformation at the other, all leading to DNA damage (Hoeijmakers, 2001; Shah et al., 2017; Shah, 2020; Kirby and Lammerding, 2018; Tubbs and Nussenzweig, 2017; Vitale et al., 2017; Reddien and Sánchez Alvarado, 2004; Wenemoser and Reddien, 2010; Lomakin et al., 2020; Irianto et al., 2017b; Mandal et al., 2011; Chen et al., 2015; Earle et al., 2020). We provide evidence of how adult stem cell migration can lead to DNA damage in vivo, thereby providing a physiological relevance to the relationship between cell migration and DNA damage of normal stem cells within a whole organism. Bringing our observations together, we propose a model where migrating cells go through a ‘migration–damage–repair–migration’ cycle as they move towards the wound site (Figure 4J).

Both ageing and oncogenic phenotypes that are commonly thought to be caused by mutations due to replicative stress may also result from genome instability incurred during cell migration. This could be a previously under-appreciated source of further genomic heterogeneity in highly invasive cancer cells that encounter tight spaces in the tissue microenvironment (Irianto et al., 2017a; Irianto et al., 2016; Vogelstein et al., 2013; Pfeifer et al., 2017), or a source of mutations in normal homeostasis and development in animals. Future work on naturally occurring MCDD will help to reveal the regulatory interplay between stem cell migration and DNA repair processes.

## Materials and methods

**Key resources table:** 

Reagent type (species) or resource	Designation	Source or reference	Identifiers	Additional information
Strain, strain background (species) (*Schmidtea mediterranea*)	Asexual clonal line CIW4	Inhouse laboratory cultured		All animals used in this study
Gene (*Schmidtea mediterranea*)	RAD51	GenBank	KM487300.1	RNAi
Gene (*Schmidtea mediterranea*)	BRCA2	GenBank	KT375435.1	RNAi
Gene (*Schmidtea mediterranea*)	ATR	Planmine	dd_Smed_v6_8754_0_1	RNAi
Gene (*Schmidtea mediterranea*)	ATM	Planmine	dd_Smed_v6_14586_0_1	RNAi
Gene (*Schmidtea mediterranea*)	FANC-J	Planmine	dd_Smed_v6_16638_0_2	RNAi
Gene (*Schmidtea mediterranea*)	PARP1	Planmine	dd_Smed_v6_10338_0_1	RNAi
Gene (*Schmidtea mediterranea*)	PARP2	Planmine	dd_Smed_v6_6154_0_1	RNAi
Gene (*Schmidtea mediterranea*)	PARP3	Planmine	dd_Smed_v6_2611_0_1	RNAi
Antibody	Anti-digoxigenin-POD, Fab fragments (rabbit polyclonal)	Sigma/Roche	# 11207733910 RRID:AB_514500	FISH (1:2000)
Antibody	Anti-fluorescein-POD, Fab fragments (rabbit polyclonal)	Sigma/Roche	#11426346910 RRID:AB_840257	FISH (1:2000)
Antibody	Anti-H3P (phosphorylated serine 10 on histone H3 (rabbit polyclonal))	Millipore	#09–797 RRID:AB_1977177	IF (1:1000)
Antibody	Anti-Poly (ADP) Ribose (PAR) monoclonal antibody (clone 10H) (mouse monoclonal)	Santacruz	#SC-56198 RRID:AB_785249	IF (1:250)
Antibody	Anti-TUDOR-1(Tud1) (rabbit polyclonal)	Aboobaker Lab Solana et al., 2009		IF (1:250)
Antibody	Goat-Anti-rabbit-HRP (goat polyclonal)	Invitrogen	#65–6120	IF (1:2000)
Antibody	Goat-Anti-mouse HRP (goat polyclonal)	Invitrogen	#62–6520	IF (1:2000)
Chemical compound, drug	Formaldehyde	EMD Millipore	FX0410-5	Used at 4% for fixing animals
Chemical compound, drug	Platinum Taq	Invitrogen	#10966026	PCR
Chemical compound, drug	Trizol	Invitrogen	#15596026	RNA isolation
Chemical compound, drug	Superscript III Reverse transcriptase	Invitrogen	#18080093	cDNA synthesis
Chemical compound, drug	Absolute qPCR mix, SYBR Green	Thermo Fisher	#AB1159A	RT-qPCR expression
Chemical compound, drug	Chloretone	Sigma	#112054	Anaesthetising worms
Chemical compound, drug	Instant ocean Sea Salt	Instant Ocean	#SS15-10	Culturing animals
Sequence-based reagent	ATR_F	Sigma	GCGCAGGAATTCAGAAACTC	dsRNA for RNAi
Sequence-based reagent	ATR_R	Sigma	GACGGTCACCGAGACCTAAA	dsRNA for RNAi
Sequence-based reagent	ATM_F	Sigma	ATTCACTGGGCCAACGTTGA	dsRNA for RNAi
Sequence-based reagent	ATM_R	Sigma	TCTTCCCTCGACACCAAACG	dsRNA for RNAi
Sequence-based reagent	BRCA2_F	Sigma	ATGGACGGGATGTGATGAGC	dsRNA for RNAi
Sequence-based reagent	BRCA2_R	Sigma	ATGCACCTTCCACGAGCAAT	dsRNA for RNAi
Sequence-based reagent	Rad51_F	Sigma (Peiris et al., 2016)	TTTGCAAGGTGGTGTTGAAA	dsRNA for RNAi
Sequence-based reagent	Rad51_R	Sigma (Peiris et al., 2016)	ATCAGCCAACCGTAACAAGG	dsRNA for RNAi
Sequence-based reagent	FancJ_F	Sigma	AGCGGAAAGGAAGACTGTCA	dsRNA for RNAi
Sequence-based reagent	FancJ_R	Sigma	TAGGCACGACTTCACTGCAC	dsRNA for RNAi
Sequence-based reagent	PARP1_F	Sigma	AACGTGCAATGCTGGAGTTT	dsRNA for RNAi
Sequence-based reagent	PARP1_R	Sigma	TCCTACCCCTTTGCAACTGT	dsRNA for RNAi
Sequence-based reagent	PARP2_F	Sigma	TGACTGGCAAGATCGTCAGA	dsRNA for RNAi
Sequence-based reagent	PARP2_R	Sigma	AGTTGTTCTTGAACCGTGCC	dsRNA for RNAi
Sequence-based reagent	PARP3_F	Sigma	AACTCTTGTGGCATGGAACC	dsRNA for RNAi
Sequence-based reagent	PARP3_R	Sigma	CGCAGAGTTCGTGAAATGAA	dsRNA for RNAi
Sequence-based reagent	Atr_qPCR_F	Sigma	ACGCGTGGTATAGGAGCGTG	qPCR
Sequence-based reagent	Atr_qPCR_R	Sigma	TATGACGGTCACCGAGACC	qPCR
Sequence-based reagent	Atm_qPCR_F	Sigma (Peiris et al., 2016)	CTGATTGGTCGGCTTTCATT	qPCR
Sequence-based reagent	Atm_qPCR_R	Sigma (Peiris et al., 2016)	AGCTAACCAATCCCCCAAAG	qPCR
Sequence-based reagent	Brca2_qPCR_F	Sigma (Peiris et al., 2016)	CAAAGAGACCCTGCTTGAGG	qPCR
Sequence-based reagent	Brca2_qPCR_R	Sigma (Peiris et al., 2016)	AGCCGGAACACAGTACCATC	qPCR
Sequence-based reagent	Rad51_qPCR_F	Sigma (Peiris et al., 2016)	ATGTCAGAATCCCGATACGC	qPCR
Sequence-based reagent	Rad51_qPCR_R	Sigma (Peiris et al., 2016)	ATCAGCCAACCGTAACAAGG	qPCR
Sequence-based reagent	FancJ_qPCR_F	Sigma	CACCAGTGGAACCTTATCTCC	qPCR
Sequence-based reagent	FancJ_qPCR_R	Sigma	GGACGGTCCGTTTCCGATGCT	qPCR
Sequence-based reagent	Parp1_qPCR_F	Sigma	CGATTCTATACAATGATGCC	qPCR
Sequence-based reagent	Parp1_qPCR_R	Sigma	CTGCTTCCATCAGTTTATAGGC	qPCR
Sequence-based reagent	Parp2_qPCR_F	Sigma	CAAGAACAACTAATTACGGTGG	qPCR
Sequence-based reagent	Parp2_qPCR_R	Sigma	GATCTCGTCGGGTAATATAG	qPCR
Sequence-based reagent	Parp3_qPCR_F	Sigma	GATATTGAAAGTACTCAAGC	qPCR
Sequence-based reagent	Parp3_qPCR_R	Sigma	CAACATCTAGCATCTTGAACC	qPCR
Software algorithm	TBLASTX	U.S. National Library of Medicine	RRID:SCR_011823	Human gene comparison
Software algorithm	BLASTX	U.S. National Library of Medicine	RRID:SCR_001653	Homology searches
Software algorithm	eggNOG.5.0	European Molecular Biology Laboratory, Hiedelberg	RRID:SCR_002456	Identify orthologs
Software algorithm	Inparanoid	http://inparanoid.sbc.su.se/cgi-bin/index.cgi	RRID:SCR_006801	Identify orthologs
Software algorithm	Planmine	MPI-CBG, Dresden (Dr. Jochen Rink)	http://planmine.mpi-cbg.de/	Identify flatworm sequences
Software algorithm	Fiji/Image-J	MPI-CBG, Dresden/National Institutes of Health (NIH)	PMID:22743772 RRID:SCR_002285	Image processing and analysis
Software algorithm	KOMET (andor)	Oxford instruments	https://andor.oxinst.com/products/komet-software/	Comet assay analysis
Software algorithm	Graphpad Prism v6	Graphpad	RRID:SCR_002798	Graphs and statistical analysis
Software algorithm	Illustrator CC	Adobe	RRID:SCR_010279	Making figures

### Planarian culture

Asexual freshwater planarians of the species *S. mediterranea* were used in this study. The culture was maintained in 0.5% instant ocean solution (which we have referred to as planarian water in this paper) and fed with organic calf liver twice a week. Planarians were starved for 7 days prior to each experiment and also throughout the duration of each experiment and cultured in the dark at 20°C.

### Gene cloning and RNAi

The sequences of *S. mediterranea* RAD51 and BRCA2 were described previously (Peiris et al., 2016). Planarian DDR genes were identified by BLAST searches against the Planmine database, leading to the identification of full-length mRNA transcripts (details in key resources table). Fragments of these genes were cloned into the pPR-T4P plasmid vector containing opposable T7 promoters (kind gift from Jochen Rink, MPI Dresden). These clones were used for in vitro transcription to synthesise dsRNA and RNA probes as previously described in Abnave et al., 2017. dsRNA was delivered via microinjection using Nanoject II apparatus (Drummond Scientific) with 3.5’’ Drummond Scientific (Harvard Apparatus) glass capillaries pulled into fine needles on a Flaming/Brown Micropipette Puller (Patterson Scientific). Worms were injected with 3 × 32 nl of dsRNA six times over 2 weeks. A 1 day gap was kept between the last injection and irradiation experiments (as described in Abnave et al., 2017). The primers used for amplification of DNA for dsRNA synthesis/RNA probes can be found in key resources table. Identification of orthologous genes across animal species was done using the Inparanoid database (O'Brien et al., 2005) (http://inparanoid.sbc.su.se/cgi-bin/index.cgi) and EggNOG database (Huerta-Cepas et al., 2019) (http://eggnogdb.embl.de). The phylogenetic tree is based on that presented by Grohme et al., 2018. We also used reciprocal blastp result against the nr database and tblastn result against each sequence. The Planmine database (Rozanski et al., 2019) was used for the identification of sequences of *S. mediterranea* and other flatworm species.

### Gamma irradiation

Animals were starved for at least 7 days and exposed to 1.5, 5, 10, 15, 20, and 30 Gy of 137 Cs gamma rays (for Figure 1A) using a GSR D1 Gsm (Gamma-Service Medical GmbH, Leipzig, Germany) gamma irradiator at a dose rate of 1.9 Gy/min. This device was also used to apply doses to whole worms before or after shielded irradiation.

### Shielded irradiation assay

Shielded irradiation was performed as previously described (Abnave et al., 2017). Worms (3–5 mm) were anesthetised in ice-cold 0.2% chloretone and aligned on 60 mm Petri dish. The Petri dish was pre-marked with a line at the bottom according to the dimension of the shield (as described in Figure 2—figure supplement 1A). The anterior tips of individual worms were aligned to keep the absolute migratory distance between the tip of head and the shielded region fixed. The Petri dish containing worms was then placed directly on top of the lead shield and irradiated from below with X-rays (225 kV, 0.5 mm AI filter, 23 Gy/min). The head and tail regions of the worms received 30 Gy, while the shielded region received less than 1.5 Gy (Figure 2—figure supplement 1B). Immediately following irradiation, the worms were placed into fresh planarian water and cultured in the dark at 20°C. For experiments involving an initial dose of gamma irradiation, worms were incubated for 15 min in planarian water before being used for shielded irradiation assay (Experiment in Figure 3A). Heads were amputated 4 days post-shielded irradiation to induce migration towards the wound (considered as 0 day post-amputation [dpa] or modified as necessary for a particular experiment [e.g. Figure 3A]). Lack of posterior cell migration in the absence of a posterior wounds allowed us to define the boundary of the shield and measure the distance migrated by stem cells in whole mount samples as previously described (Abnave et al., 2017).

### COMET assay in planarians

Frosted microscope slides were coated with 700 μl of 1% normal-melting-point agarose (NMPA) in 1× PBS to make a uniform layer and dried overnight at 55°C. Worm fragments were gently diced to minimise any mechanical stress to the cells. The tissue pieces were digested using Papain (15 U/ml) for 1 hr at 25°C. Pieces were mechanically dissociated using a P1000 pipette to form a single-cell suspension and filtered through 100 μm and then 35 μm cell strainers (BD Falcon). Ten thousand dissociated single cells were re-suspended in 80 μl of CMFHE^2+^, and an equal amount of 1.5% low-melting-point agarose was added and mixed. Forty microlitres of the cell-agarose suspension was added onto an NMPA-coated slide and allowed to solidify at 4°C. Slides were incubated overnight (~15 hr) in a coplin jar at 4°C with lysing solution ([2.5 M NaCl, 100 mM EDTA, 10 mM trizma base, NaOH added to pH 10.0] and freshly added 1% triton x-100). This solution was then replaced with a neutralisation buffer (0.4 M Tris base in dH_2_O, pH to 7.5) for 15 min at 4°C. Following this, the neutralisation buffer was then removed, and the slides were placed into an electrophoresis chamber at 4°C filled with freshly prepared 1× electrophoresis buffer (300 mM NaOH and 1 mM EDTA in dH_2_O) at 4°C. The slides were allowed to equilibrate for 15 min followed by an alkaline electrophoresis at 20 V for 20 min at 4°C. Next, slides were transferred back into the coplin jar and equilibrated for 5 min in neutralisation buffer. The slides were stained with SYBR Green I (1:10,000 dilution) in freshly prepared 1× TE buffer (10 mM Tris–HCl and 1 mM EDTA, pH 7.5). For long-term storage, the slides were fixed with cold 100% ethanol for 5 min and dried. After drying, 50 comets per slides were analysed using KOMET software (Andor), and the percentage of tail DNA was measured after different doses of gamma irradiation or from ‘shielded’ regions or ‘migrating’ regions both with and without migrating stem cells (i.e. with or without wounding). The shielded and unshielded areas were amputated away from each other based on visual estimation. In order to estimate the accuracy of the dissected areas, we performed Comet assay or *smedwi-*FISH on the same animals, such that if a piece (shielded) is used for Comet assay, then the corresponding part (unshielded migratory) is used for *smedwi-1* FISH to assure the accuracy of amputation.

Each FISH was performed in three replicates (InM1, InM2, InM3) with three worms per batch (‘In’ or ‘Wo’ corresponding to Intact or Wounded. ‘M’ and ‘S’ corresponds to Migratory tissue or Shielded tissue). The corresponding tissues from these worms were pooled by experimental groups for use in the COMET assay. Individual replicates were analyzed for the presence of *smedwi-1* cells to confirm the accuracy of separating shielded vs unshielded areas.

For comet assay, cells were embedded into four to six slides/replicate, and 50 comets were randomly scored from each slide to a total of 200–300 comets analyzed per condition per replicate. Each of these replicates are shown in Figure 2—figure supplement 3A–G, with the detailed schematic on the experimental plan and subsequent analysis.

The extensive DNA fragmentation that occurs during apoptosis does not show comet-like structures; hence, comet tails are representative of DNA breaks in remaining cells. Fragmented DNA in an apoptotic nuclei is very small (size of a nucleosome oligomer) and would generally disappears completely by diffusion in the gel during lysis and/or electrophoresis and are mostly seen as diffuse spheroid or like a halo around the nucleoid-head in comet images and does not show a structure that resembles comet-tail. (For interpretation of Comet assay results, see Lorenzo et al., 2013; Collins, 2004.)

### In situ hybridisation and immunostaining

All fluorescence in situ hybridisation (FISH) experiments are performed against a particular mRNA of interest, thereby allow detection of the mRNA expression. FISH was performed as described in King and Newmark, 2013 and previously reported sequences were used for riboprobe synthesis of smedwi-1 (Abnave et al., 2017). The antibodies used for immunostaining were anti-H3P (phosphorylated serine 10 on histone H3; Millipore; 09–797; 1:1000 dilution Abnave et al., 2017), anti-poly (ADP) ribose (Shibata et al., 2016) (PAR) monoclonal antibody (1:250) (Santacruz, clone 10H), and anti-TUD1 (1:250 dilution, based on Solana et al., 2009). Anti-rabbit-HRP (H3P and TUD-1) and anti-mouse-HRP (PAR) (1:2000 dilution) secondary antibodies were used followed by tyramide signal amplification for FISH and immunostaining as described in King and Newmark, 2013.

### Sectioning of planarian worms

Planarians were killed in 2% HCl and Holtfreter’s solution and fixed with 4% formaldehyde for 2 hr. The worms were then washed in PBSTx (0.3% triton-X) and dehydrated with an increasing gradient of methanol washes and stored at −20°C. The following day worms were re-hydrated with a decreasing gradient of methanol and PBS, Xylene washes (two washes of 7 min each) and placed in molten paraffin for 1 hr. Individual worms were then aligned (sagittal or transverse) in paraffin moulds, trimmed, and sliced into 10 μm sections using a microtome. Individual ribbons of planarian sections were placed in a 37°C water bath and aligned to have the entire worm on each poly-lysine-coated slide.

### Immunostaining on paraffinised sections

Planarian sections were deparaffinised using xylene substitute (two washes of 7 min each) and washed with PBS-Tx0.3 (0.3% triton-X). The sections were subjected to antigen retrieval with Trilogy (Cell Marque) at 90°C. The slides were then fixed in 4% formaldehyde for 15 min followed by two washes of PBS-Tx0.5 (0.5% triton-X) for 30 min and transferred to a blocking solution (0.5% BSA and PBS-Tx0.5). One hundred and fifty microlitres of primary antibody (diluted in blocking solution) was added to individual slides, and a parafilm was placed on top for uniform spreading of the antibody solution. After an overnight antibody incubation at 4°C, the slides were washed with alternating changes of PBS-Tx0.5 and PBS + 0.1% tween-20. The secondary antibodies were diluted in blocking solution and incubated overnight. The slides were washed again with alternating changes of PBS-Tx0.5 and PBS + 0.1% tween-20. After two 10 min washes, slides were developed with Tyramide/other fluorophores. For double immunostaining, sodium-azide based peroxide inactivation was performed after the development of each antibody. After two 10 min washes with PBS-TW, slides were stained with Hoechst for nuclear staining overnight. Slides were mounted and imaged using a 100× oil objective lens in an Olympus FV1000 confocal microscope with the appropriate fluorescent lasers.

### Image processing and data analysis

Whole worm confocal imaging was done with Olympus FV1000 and taken as Z-stacks (slices of 4 μm each D/V axis) that were stitched and then processed as a maximum projection using Fiji software (https://fiji.sc/). All measurements and quantifications were done with Fiji (using cell counter plug in) and normalised to the area. Images in Figure 2A,B, i-iv are single confocal stacks (0.32 μm) taken with a Zeiss 880 Airyscan microscope using a 63× oil objective lens and manually cropped into individual cells for counting in Fiji software. Nuclear aspect ratio was measured taking the ratio of the length and the width of the nucleus (Chen et al., 2015). Images were then processed and cropped before pseudo-colouring the signals in Fiji. The background is set to black for better visualisation, and all figures are prepared using Adobe Illustrator v6 with colour combinations in CMYK format to make scientific figures accessible to people with colour-blindness. Quantification of PAR fluorescence was performed in Fiji using the ‘mean fluorescence analysis’ tool and normalised to the nuclear area using the Hoechst signal. Total PAR fluorescence was measured from all the nuclei, with high intensity of perinuclear TUD1 staining used to determine Tudor-1-positive stem cells (Solana et al., 2009).

### Quantitative RT-qPCR

Total RNA from samples of three to five worms were extracted with Trizol reagent (Invitrogen) according to the manufacturer's instructions for each of two biological replicates for each RNAi condition. RNA was treated with TURBO DNase (Ambion). First-strand cDNAs were synthesised with SuperScript III reverse transcriptase (Invitrogen) and qRT-PCR experiments used the Absolute qPCR SYBR Green Master Mix (Thermo Scientific). Experiments were performed on two biological replicates per RNAi condition. Each biological replicates was technically replicated three times, with each technical replicate consisting of three replicate amplification reactions. Primers for DDR gene are listed in the key resources table. *Smed-ef-2* was used for normalisation using primers described previously (Solana et al., 2013).

### Statistical analysis

Results are expressed as mean ± standard deviation (SD). Statistical analyses were performed using GraphPad Prism version 6.0 (https://www.graphpad.com/). Student’s t-test and Tukey’s multiple comparison test were used for statistical significance at p<0.05. Exact p-values are reported for all experiments.

## Data Availability

All data generated or analysed during this study are included in the manuscript and supporting files.

## Additional files

Transparent reporting form

## References

[bib1] Abnave P, Aboukhatwa E, Kosaka N, Thompson J, Hill MA, Aboobaker AA (2017). Epithelial-mesenchymal transition transcription factors control pluripotent adult stem cell migration *in vivo* in planarians. Development.

[bib2] Aboobaker AA (2011). Planarian stem cells: a simple paradigm for regeneration. Trends in Cell Biology.

[bib3] Adams PD, Jasper H, Rudolph KL (2015). Aging-Induced stem cell mutations as drivers for disease and Cancer. Cell Stem Cell.

[bib4] Bennett RR, Pfeifer CR, Irianto J, Xia Y, Discher DE, Liu AJ (2017). Elastic-Fluid model for DNA damage and mutation from nuclear fluid segregation due to cell migration. Biophysical Journal.

[bib5] Brandl H, Moon H, Vila-Farré M, Liu SY, Henry I, Rink JC (2016). PlanMine--a mineable resource of planarian biology and biodiversity. Nucleic Acids Research.

[bib6] Chen B, Co C, Ho CC (2015). Cell shape dependent regulation of nuclear morphology. Biomaterials.

[bib7] Collins AR (2004). The comet assay for DNA damage and repair: principles, applications, and limitations. Molecular Biotechnology.

[bib8] Dattani A, Kao D, Mihaylova Y, Abnave P, Hughes S, Lai A, Sahu S, Aboobaker AA (2018). Epigenetic analyses of planarian stem cells demonstrate conservation of bivalent histone modifications in animal stem cells. Genome Research.

[bib9] Denais CM, Gilbert RM, Isermann P, McGregor AL, te Lindert M, Weigelin B, Davidson PM, Friedl P, Wolf K, Lammerding J (2016). Nuclear envelope rupture and repair during Cancer cell migration. Science.

[bib10] Earle AJ, Kirby TJ, Fedorchak GR, Isermann P, Patel J, Iruvanti S, Moore SA, Bonne G, Wallrath LL, Lammerding J (2020). Mutant lamins cause nuclear envelope rupture and DNA damage in skeletal muscle cells. Nature Materials.

[bib11] Gehrke AR, Srivastava M (2016). Neoblasts and the evolution of whole-body regeneration. Current Opinion in Genetics & Development.

[bib12] Goodell MA, Rando TA (2015). Stem cells and healthy aging. Science.

[bib13] Grohme MA, Schloissnig S, Rozanski A, Pippel M, Young GR, Winkler S, Brandl H, Henry I, Dahl A, Powell S, Hiller M, Myers E, Rink JC (2018). The genome of *Schmidtea mediterranea* and the evolution of core cellular mechanisms. Nature.

[bib14] Guedelhoefer OC, Sánchez Alvarado A (2012). Amputation induces stem cell mobilization to sites of injury during planarian regeneration. Development.

[bib15] Hoeijmakers JH (2001). Genome maintenance mechanisms for preventing cancer. Nature.

[bib16] Huerta-Cepas J, Szklarczyk D, Heller D, Hernández-Plaza A, Forslund SK, Cook H, Mende DR, Letunic I, Rattei T, Jensen LJ, von Mering C, Bork P (2019). eggNOG 5.0: a hierarchical, functionally and phylogenetically annotated orthology resource based on 5090 organisms and 2502 viruses. Nucleic Acids Research.

[bib17] Irianto J, Pfeifer CR, Bennett RR, Xia Y, Ivanovska IL, Liu AJ, Greenberg RA, Discher DE (2016). Nuclear constriction segregates mobile nuclear proteins away from chromatin. Molecular Biology of the Cell.

[bib18] Irianto J, Xia Y, Pfeifer CR, Athirasala A, Ji J, Alvey C, Tewari M, Bennett RR, Harding SM, Liu AJ, Greenberg RA, Discher DE (2017a). DNA damage follows repair factor depletion and portends genome variation in Cancer cells after pore migration. Current Biology.

[bib19] Irianto J, Xia Y, Pfeifer CR, Greenberg RA, Discher DE (2017b). As a nucleus enters a small pore, chromatin stretches and maintains integrity, even with DNA breaks. Biophysical Journal.

[bib20] Jackson SP, Bartek J (2009). The DNA-damage response in human biology and disease. Nature.

[bib21] Jeggo PA, Pearl LH, Carr AM (2016). DNA repair, genome stability and Cancer: a historical perspective. Nature Reviews Cancer.

[bib22] King RS, Newmark PA (2013). In situ hybridization protocol for enhanced detection of gene expression in the planarian *Schmidtea mediterranea*. BMC Developmental Biology.

[bib23] Kirby TJ, Lammerding J (2018). Emerging views of the nucleus as a cellular mechanosensor. Nature Cell Biology.

[bib24] Lei K, Thi-Kim Vu H, Mohan RD, McKinney SA, Seidel CW, Alexander R, Gotting K, Workman JL, Sánchez Alvarado A (2016). Egf signaling directs neoblast repopulation by regulating asymmetric cell division in planarians. Developmental Cell.

[bib25] Lomakin AJ, Cattin CJ, Cuvelier D, Alraies Z, Molina M, Nader GPF, Srivastava N, Sáez PJ, Garcia-Arcos JM, Zhitnyak IY, Bhargava A, Driscoll MK, Welf ES, Fiolka R, Petrie RJ, De Silva NS, González-Granado JM, Manel N, Lennon-Duménil AM, Müller DJ, Piel M (2020). The nucleus acts as a ruler tailoring cell responses to spatial constraints. Science.

[bib26] Lorenzo Y, Costa S, Collins AR, Azqueta A (2013). The comet assay, DNA damage, DNA repair and cytotoxicity: hedgehogs are not always dead. Mutagenesis.

[bib27] Mandal PK, Blanpain C, Rossi DJ (2011). DNA damage response in adult stem cells: pathways and consequences. Nature Reviews Molecular Cell Biology.

[bib28] Nader GPF (2020). Compromised nuclear envelope integrity drives tumor cell invasion. bioRxiv.

[bib29] O'Brien KP, Remm M, Sonnhammer EL (2005). Inparanoid: a comprehensive database of eukaryotic orthologs. Nucleic Acids Research.

[bib30] Peiris TH, Ramirez D, Barghouth PG, Ofoha U, Davidian D, Weckerle F, Oviedo NJ (2016). Regional signals in the planarian body guide stem cell fate in the presence of genomic instability. Development.

[bib31] Pfeifer CR, Alvey CM, Irianto J, Discher DE (2017). Genome variation across cancers scales with tissue stiffness – An invasion-mutation mechanism and implications for immune cell infiltration. Current Opinion in Systems Biology.

[bib32] Pfeifer CR, Xia Y, Zhu K, Liu D, Irianto J, García VMM, Millán LMS, Niese B, Harding S, Deviri D, Greenberg RA, Discher DE (2018). Constricted migration increases DNA damage and independently represses cell cycle. Molecular Biology of the Cell.

[bib33] Raab M, Gentili M, de Belly H, Thiam HR, Vargas P, Jimenez AJ, Lautenschlaeger F, Voituriez R, Lennon-Duménil AM, Manel N, Piel M (2016). ESCRT III repairs nuclear envelope ruptures during cell migration to limit DNA damage and cell death. Science.

[bib34] Reddien PW, Sánchez Alvarado A (2004). Fundamentals of planarian regeneration. Annual Review of Cell and Developmental Biology.

[bib35] Rink JC (2013). Stem cell systems and regeneration in Planaria. Development Genes and Evolution.

[bib36] Robb SM, Ross E, Sánchez Alvarado A (2008). SmedGD: the *Schmidtea mediterranea* genome database. Nucleic Acids Research.

[bib37] Rossi DJ, Jamieson CH, Weissman IL (2008). Stems cells and the pathways to aging and Cancer. Cell.

[bib38] Rozanski A, Moon H, Brandl H, Martín-Durán JM, Grohme MA, Hüttner K, Bartscherer K, Henry I, Rink JC (2019). PlanMine 3.0-improvements to a mineable resource of flatworm biology and biodiversity. Nucleic acids research.

[bib39] Sahu S, Dattani A, Aboobaker AA (2017). Secrets from immortal worms: what can we learn about biological ageing from the planarian model system?. Seminars in Cell & Developmental Biology.

[bib40] Shah P, Wolf K, Lammerding J (2017). Bursting the bubble - Nuclear envelope rupture as a path to genomic instability?. Trends in Cell Biology.

[bib41] Shah P (2020). Nuclear deformation causes DNA damage by increasing replication stress. bioRxiv.

[bib42] Shibata N, Kashima M, Ishiko T, Nishimura O, Rouhana L, Misaki K, Yonemura S, Saito K, Siomi H, Siomi MC, Agata K (2016). Inheritance of a nuclear PIWI from pluripotent stem cells by somatic descendants ensures differentiation by silencing transposons in planarian. Developmental Cell.

[bib43] Smith LR, Irianto J, Xia Y, Pfeifer CR, Discher DE (2019). Constricted migration modulates stem cell differentiation. Molecular Biology of the Cell.

[bib44] Solana J, Lasko P, Romero R (2009). Spoltud-1 is a chromatoid body component required for planarian long-term stem cell self-renewal. Developmental Biology.

[bib45] Solana J, Gamberi C, Mihaylova Y, Grosswendt S, Chen C, Lasko P, Rajewsky N, Aboobaker AA (2013). The CCR4-NOT complex mediates deadenylation and degradation of stem cell mRNAs and promotes planarian stem cell differentiation. PLOS Genetics.

[bib46] Sperka T, Wang J, Rudolph KL (2012). DNA damage checkpoints in stem cells, ageing and Cancer. Nature Reviews Molecular Cell Biology.

[bib47] Tubbs A, Nussenzweig A (2017). Endogenous DNA Damage as a Source of Genomic Instability in Cancer. Cell.

[bib48] Vitale I, Manic G, De Maria R, Kroemer G, Galluzzi L (2017). DNA Damage in Stem Cells. Molecular cell.

[bib49] Vogelstein B, Papadopoulos N, Velculescu VE, Zhou S, Diaz LA, Kinzler KW (2013). Cancer genome landscapes. Science.

[bib50] Wagner DE, Wang IE, Reddien PW (2011). Clonogenic neoblasts are pluripotent adult stem cells that underlie planarian regeneration. Science.

[bib51] Wagner DE, Ho JJ, Reddien PW (2012). Genetic regulators of a pluripotent adult stem cell system in planarians identified by RNAi and clonal analysis. Cell stem cell.

[bib52] Wenemoser D, Reddien PW (2010). Planarian regeneration involves distinct stem cell responses to wounds and tissue absence. Developmental biology.

[bib53] Wouters A, Ploem J-P, Langie SAS, Artois T, Aboobaker A, Smeets K (2020). Regenerative responses following DNA damage – β-catenin mediates head regrowth in the planarian *Schmidtea mediterranea*. Journal of Cell Science.

[bib54] Wurtzel O, Cote LE, Poirier A, Satija R, Regev A, Reddien PW (2015). A generic and Cell-Type-Specific wound response precedes regeneration in planarians. Developmental Cell.

